# Editorial: Neuropeptide GPCRs in neuroendocrinology, Volume II

**DOI:** 10.3389/fendo.2023.1219530

**Published:** 2023-06-21

**Authors:** Hubert Vaudry, Liliane Schoofs, Olivier Civelli, Masayasu Kojima

**Affiliations:** ^1^ Institute of Biomedical Research and Innovation, University of Rouen Normandy, Mont-Saint-Aignan, France; ^2^ Department of Biology, KU Leuven, Leuven, Belgium; ^3^ Department of Pharmaceutical Sciences, University of California, Irvine, Irvine, CA, United States; ^4^ Institute of Life Science, Kurume University, Fukuoka, Japan

**Keywords:** neuropeptides, G protein-coupled receptors, biologically active peptides, signaling mechanisms, neuroendocrine communication

Neuropeptides are the largest and most diverse class of neuromediators in the central and peripheral nervous systems. They play multiple roles in the control of various biological functions including feeding, reproduction, development, growth, learning, nociception, stress coping, thermoregulation, osmoregulation, and a vast array of behaviors. Most neuropeptides exert their activities through G protein-coupled receptors (GPCRs), which represent the largest family of cell membrane receptors. Neuropeptide signaling is phylogenetically conserved throughout the animal kingdom from cnidarians to mammals. Not surprisingly, neuropeptides and their GPCRs are implicated in a number of pathologies such as obesity, infertility, stunting, pain, narcolepsy, diabetes insipidus, gastrointestinal diseases and mood disorders. Therefore, drugs targeting neuropeptide GPCRs have strong potential for the development of novel therapeutic agents.

To celebrate the 10^th^ anniversary of the Nobel Prize awarded to Robert J. Lefkowitz and Brian K. Kobilka for their seminal discoveries of the inner working of GPCRs, this Research Topic is aimed at gathering a bouquet of 27 review papers and original articles, written by prominent scientists in this fast-evolving field, that illustrate the crucial role of neuropeptide GPCRs in neuroendocrinology.

Ghrelin is a 28-amino acid acylated peptide, initially isolated from the rat stomach, that stimulates growth hormone (GH) release from pituitary cells through activation of a GPCR, the GH secretagogue receptor (GHSR) (1). Central and peripheral administration of ghrelin stimulates food intake and increases body weight (2). Hassouna et al. now show that, in adult female, but not in male mice, preproghrelin gene deletion markedly attenuates pulsatile GH secretion. Surprisingly, however, in *Ghrl^-/-^* mice, food consumption and body weight are unaltered. The lateral parabrachial nucleus (lPBN) includes a population of anorexic neurons (3) and abundantly expresses GHSR mRNA (4). Le May et al. report that selective silencing of GHSR-positive cells of the lPBN inhibits food intake and reduces fat weight, indicating that these GHSR-expressing cells are involved in hyperphagia and weight gain. It has recently been reported that a peptide called liver-expressed antimicrobial peptide 2 (LEAP-2) acts as a natural antagonist of GHSR1a (5). The review by Lu et al. discusses the mechanism of action of LEAP-2 both as an inverse agonist and antagonist of GHSR1a, and the potential applications of this novel peptide in various pathologies including obesity.

Vasoactive intestinal peptide (VIP) and pituitary adenylate cyclase activating polypeptide (PACAP) are two related peptides that act via three GPCRs i.e. the PACAP-specific receptor PAC1 and the VIP/PACAP mutual receptors VPAC1 and VPAC2 (6). Consistent with the widespread distribution of these receptors in the central nervous system (CNS) and in peripheral organs, VIP and PACAP exert a large array of biological activities (7). Ago et al. review the evidence linking VIP2 microduplication to schizophrenia and other psychiatric disorders. They propose that excessive VPAC2 signaling disrupts maturation of certain brain structures including the prefrontal cortex. It has been previously reported that PACAP knock-out (*Pacap* ^-/-^) mice exhibit behavioral abnormalities including hyperlocomotor activity and deficit in prepulse inhibition (8) that are reversed by 5-HT_2A_ receptor antagonists (9). Here, Hayata-Takano et al. show that PACAP-PAC1 signaling induces internalization of 5-HT_2A_ receptors in transfected cells and that *Pacap* ^-/-^ mice exhibit an increase of 5HT_2A_ levels on cell membranes in the frontal cortex, suggesting that the PAC1 receptor could be a target for the treatment of some psychiatric disorders. VIP/VPAC2 signaling plays a crucial role in the control of the circadian clock in the suprachiasmatic nucleus (SCN) (10). Since thyroid cells possess a circadian clock (11), Georg et al. have investigated the possible impact of VIP/VPAC2 signaling in the expression of thyroid clock genes. The data indicate that the thyroid clock is largely independent from the master SCN clock. The neurotrophic and neuroprotective effects of VIP and PACAP are partly mediated by a glial protein termed activity-dependent neuroprotective protein (ADNP) (12). In an Opinion paper, Gozes and Shazman discuss the possible involvement of ADNP gene mutations at protease cleavage sites in autism and Alzheimer’s disease.

Gonadotropin inhibitory hormone (GnIH) is a C-terminally amidated dodecapeptide initially isolated from the quail brain on the basis of its ability to inhibit gonadotropin release (13). The mammalian orthologue of GnIH is generally called RFRP-3. The effects of GnIH and its orthologues are mediated via activation of GPR147, also named GnIH-R (14). The review by Bédécarrats et al. summarizes the multiple actions of the GnIH/GnIH-R signaling system not only on reproductive functions but also on the regulation of energy homeostasis, stress response and thyroid hormone secretion. A sister review by Teo et al. focuses on the involvement of GnIH on biological rhythms, stress response and social behaviors. Taken together, these review articles illustrate the numerous activities of this fascinating peptide.

Melanocortin receptors (MCRs) constitute a family of five GPCRs with multiple physiological functions (15). The activity of MCRs is regulated by two melanocortin receptor accessory proteins, MRAP1 and MRAP2 that form heterodimers with MC2R (16) and possibly with MC3R and MC4R (17). In a hypothesis and theory article, Dores and Chapa describe the phylogenetic evolution of MC2R and its essential accessory player MRAP1. Since MRAPs function as antiparalelled homodimers, Wang et al. have investigated the internal symmetry of the MRAP2 homodimer. Their study reveals the importance of the orientation of the various domains of MRAP2 in the activity of MC4R.

The oxytocin (OT) and vasotocin (VT) genes derive from a common ancestral gene that existed before the emergence of vertebrates (18). The actions of OT and VT are mediated through a series of GPCRs (OTR and VTR) whose genes also arose from a common ancestor (19). Ocampo Daza et al. have conducted synteny analyses to elucidate the phylogenetic history of OTR and VTR in jawed vertebrates. Their data led them to recommend a rational nomenclature for OTR and VTR genes that differs from that recently proposed by Theofanopoulou et al. (20).

Apelin is a 36-amino acid peptide that counteracts the antidiuretic action of vasopressin (21). The apelin receptor APJ and the angiotensin receptor AT1 are two GPCRs which display 31% sequence identify (22). The review by Girault-Sotias et al. analyses the opposite actions of apelin and vasopressin in the control of diuresis and discusses the therapeutic potential of apelin agonists for the treatment of the syndrome of inappropriate antidiuresis.

Motilin (MLN) is a 22-amino acid peptide, isolated half a century ago from the porcine gastro-intestinal tract (23), that acts via activation of a GPCR called MLN-R. In a comparative perspective, Kitazawa and Kaiya review the current knowledge regarding the structure, distribution and biological activities of MLN and MLN-R in various vertebrate species from fish to mammals.

Bombesin (Bn) is a member of a family of bioactive peptides that also includes neuromedin B (NMB) and gastrin-releasing peptide (GRP) (24). These peptides act though three types of GPCRs i.e. the NMB receptor (NMBR), the GRP receptor (GRPR) and the Bn-receptor subtype 3 (BRS-3) now renamed by NC-IUPHAR BB1, BB2 and BB3, respectively (25). Moody et al. recapitulate the evidence that Bn-related peptides and their receptors are frequently overexpressed in a number of tumor cells, suggesting their potential for imaging and/or targeted therapy of neural tumors.

Corticotropin-releasing hormone (CRH) was initially characterized in the hypothalamus based on its ability to stimulate the release of adrenocorticotropin from pituitary corticotrope cells (26). It was later reported that CRH and/or CRH-related peptides are also present in peripheral organs, notably in the skin, immune system and reproductive organs (27). In their mini-review, Kassotaki et al. make a focus on the role of placenta CRH on fetal neurodevelopment and the control of the length of pregnancy.

Neuromedin U (NMU) and neuromedin S (NMS) are two structurally related peptides whose actions are mediated by two mutual GPCRs, i.e. NMUR1 and NMUR2 (28). Malendowicz and Rucinski provide a comprehensive review on the anatomical distribution of NMU, NMS and their receptors, and the biological and pharmacological activities of these peptidergic systems.

Orphanin FQ/nociceptin is a 17-amino acid peptide identified, via a reverse pharmacology approach, as the ligand of a GPCR that displays sequence similarity to opioid receptors (29, 30). This GPCR, now named NOP receptor, mediates pain transmission and is involved in various physiological and behavioral effects notably on locomotor activity (31). In order to develop novel analgesic compounds, Azevedo Neto et al. investigated the differential effects of NOP biased agonists on nociception vs locomotion. In normal mice, none of the NOP agonists tested exhibit functional selectivity on antinociception vs motor impairment.

Nesfatin-1, an 82-amino acid polypeptide derived from the processing of nucleobindin-2, was initially characterized for its anorexigenic activity (32). Since then, nesfatin-1 has been found to exert pleiotropic effects in the CNS and in peripheral organs (33). Here, Rupp et al. provide a comprehensive review of the multiple neuroendocrine systems and signaling pathways recruited by nesfatin-1. They point out the urgent necessity of identifying the nesfatin-1 receptor which, so far, remained elusive.

Pulsatile release of gonadotropin-releasing hormone (GnRH) is essential for normal reproductive functions (34). Uenoyama et al. describe the pivotal role of a set of neurons expressing kisspeptin, neurokinin B and dynorphin (KNDy neurons), located in the arcuate nucleus of the hypothalamus, in the control of episodic and surge secretion of GnRH. They also call attention to species and sex differences in the functioning of this GnRH pulse generator.

Processing of the neurotensin (NTS) precursor can generate a mature form of 13 amino acids and an extended form of 163 amino acids called long form NTS (LF NTS) (35). Elevated levels of LF NTS in plasma predict the incidence of diabetes and cardiovascular disease in the elderly population (36). Wu et al. have thus developed a monoclonal antibody against LF NTS. Their results show that, in mice subjected to high-fat diet, this antibody has the potential to reduce body weight and adipocyte volume.

Prokineticins (PKs) are two secreted polypeptides involved in the control of several neuroendocrine functions including reproduction, feeding behavior and circadian rhythms. The effects of PKs are mediated through activation of two GPCRs designated PKR1 and PKR2 (37). Verdinez and Sebag have identified two N-linked glycosylation positions within the N-terminal domain of PKR2 which are essential for its plasma membrane targeting and Gαs signaling.

The hypothalamo-pituitary-thyroid axis (HPT) plays a pivotal role in the control of energy homeostasis. The activity of the HPT is primarily regulated by the neuropeptide thyrotropin-releasing hormone (TRH) and its cognate GPCR expressed by pituitary thyrotrope cells (38). In their review article, Parra-Mones de Oca et al. focus on sex dimorphism in the control of the HPT activity not only resulting from sex steroids but also from differences in diet, physical activity and differential response to stress.

It is now widely accepted that many neuropeptide GPCRs have an ancient evolutionary origin and that several vertebrate GPCRs have orthologs in protostomian phyla, such as arthropods, nematodes, mollusks and annelids, among others (39). The evolutionary investigation of Li et al. shows the presence of galanin receptor (GALR)-like genes in a cephalopod mollusk and challenges the widely accepted paradigm that allatostatin-A (AST-A)/buccalin receptors are the orthologues of vertebrate GALRs in protostomes. The data further reveal that the three allatostatin peptide-receptor systems have a broad tissue distribution in bivalves and that the allatostatin-C neuropeptide system might be involved in the animal’s immune response.

Alexander et al. investigated pigment dispersing neuropeptide hormones (PDH) in the crustacean model *Carcinus maenas*, and found that 4 PDH isoforms preferentially activate two distinct PDH receptors. In addition, the study unveils a previously undescribed neurohaemal area in one of the eyestalk retractor muscles of the crab, likely to be involved in photic adaptation. The anatomical distribution of each of the four PDH neuropeptides and their GPCRs suggests distinct functions as secreted hormones and/or neuromodulators.

In insects, various hormones act upon the Malpighian tubules via a variety of GPCRs linked to second messenger systems that influence ion transporters and aquaporins; thereby regulating fluid secretion. The study of Orchard et al. reviews the current knowledge on the neuroendocrine control of diuresis and provides the reader with new insights from an in-depth transcriptome analysis of the Malpighian tubules of fed and unfed Rhodnius prolixus (kissing bug). Of particular interest is the presence of GPCR transcripts for which the role in Malpighian tubule physiology is currently unknown. As such, this study illustrates that Malpighian tubules are much more than transporting epithelia, hereby paving the way for future GPCR research.

Since about half of the most sold drugs for humans act on GPCRs, neuropeptide GPCRs have been accepted as highly druggable targets and this drug discovery potential is being extended to alternative insect pest and nematode-control strategies (40). Parasitic nematodes cause substantial morbidity and mortality in animals and people and major losses to food production. Atkinson et al. thus made use of elegant *in silico* approaches to develop a nematode drug target prioritization pipeline that highlights the most promising nematode neuropeptide GPCRs as candidate targets for parasitic control.

In summary, 27 articles addressing a variety of facets of neuropeptide GPCRs are enclosed in the present Research Topic. This collection of papers illustrates the multiple functions and therapeutical applications of neuropeptide GPCRs in neuroendocrinology. It also highlights the challenges that remain to be taken up for the next decade. It is our hope that the readers will enjoy reading these papers, and that this Research Topic will become a major set of references for all researchers involved in this rapidly expanding field.

## Author contributions

HV and LS wrote the first draft of the manuscript. OC and MK provided revisions that were incorporated by HV. All authors have read and approved the final manuscript.
